# Transdiagnostic Symptom Dimensions in Individuals at Ultra‐High Risk for Psychosis: Towards Dimensional Representations of Pluripotent Risk

**DOI:** 10.1111/eip.70086

**Published:** 2025-08-21

**Authors:** Julia C. C. Schulte‐Strathaus, Christian Rauschenberg, Isabell Paetzold, Diego Quattrone, Jessica Hartmann, Paul Amminger, Hok Pan Yuen, Patrick D. McGorry, Barnaby Nelson, Ulrich Reininghaus

**Affiliations:** ^1^ Department of Public Mental Health Central Institute of Mental Health, Medical Faculty Mannheim, Heidelberg University Mannheim Germany; ^2^ Social, Genetic and Developmental Psychiatry Centre, Institute of Psychiatry, King's College London London UK; ^3^ National Institute for Health Research (NIHR) Mental Health Biomedical Research Centre at South London and Maudsley NHS Foundation Trust and King's College London London UK; ^4^ South London and Maudsley NHS Mental Health Foundation Trust London UK; ^5^ Orygen Melbourne Victoria Australia; ^6^ Centre for Youth Mental Health, The University of Melbourne Melbourne Victoria Australia; ^7^ ESRC Centre for Society and Mental Health, King's College London London UK; ^8^ Centre for Epidemiology and Public Health, Health Service and Population Research Department Institute of Psychiatry, Psychology & Neuroscience, King's College London London UK

**Keywords:** item factor analysis, psychopathology, schizophrenia, transdiagnostic phenotypes

## Abstract

**Intro:**

Research has shifted focus from categorical to dimensional conceptualisations of mental health conditions. This is supported by high overlap between disorders, particularly psychosis spectrum and affective disorders, which cuts across traditional diagnostic boundaries. While there is evidence for a general factor of psychopathology in individuals with schizophrenia, schizoaffective disorder, and psychotic bipolar I disorder, transdiagnostic dimensions of psychopathology have not been investigated in young individuals at ultra‐high risk for psychosis, that is, youth with pluripotent symptom patterns preceding first‐episode psychosis. The current study sought to investigate (1) whether there is a general dimension underlying psychopathological symptoms in individuals and (2) whether the formation of specific symptom dimensions (i.e., positive symptoms, negative symptoms, affect, and activation) is justified alongside a general dimension.

**Methods:**

Item factor analyses were conducted on symptom ratings of the Brief Psychiatric Rating Scale at baseline in the Staged Treatment in Early Psychosis (STEP) trial.

**Results:**

In total, 342 UHR participants were included. A bifactor model with one general symptom dimension and four specific factors (positive symptoms, negative symptoms, affect, and activation) yielded the best relative model fit and interpretability when compared to uni‐ and multidimensional models, albeit absolute model fit statistics provided no strong evidence to support this finding. However, model stability and interpretability tentatively suggest it can help tell apart pluripotent and domain‐specific symptom patterns.

**Conclusion:**

Findings cautiously suggest that the BPRS may index both a general dimension and several more specific dimensions, reflecting pluripotency underlying psychological features in the sample. A ‘common ground’ across the risk syndrome in this heterogeneous sample, with various comorbidities, would lend further evidence to support the notion of a transdiagnostic phenotype in youth.

## 1 Introduction

Research increasingly focuses on dimensional conceptualisations of mental health. Emerging evidence on transdiagnostic symptom dimensions and phenotypes (Van Os and Linscott 2012; Van Os and Reininghaus 2016) challenges categories with polythetic criteria (e.g., DSM‐5 [American Psychiatric Association 2013] and ICD‐10 [World Health Organization 1993]), which may not adequately capture the continuum on which psychopathology exists (Clark et al. 2017; Haslam et al. 2012, 2020; Shah et al. 2020; Waszczuk et al. 2023; Wright et al. 2013). High comorbidity rates that cut across traditional diagnostic boundaries question the distinctiveness and validity of these diagnoses (Clark et al. 2017). Moreover, within‐diagnosis symptom heterogeneity may reflect discrete, non‐overlapping mental health conditions (Galatzer‐Levy and Bryant 2013) and correspond to different prognoses, treatments, and outcomes (Hasler et al. 2004; Shackman and Fox 2018; Zimmerman et al. 2015).

Alternative frameworks (Insel et al. 2010; Kotov et al. 2017; Ruggero et al. 2019) propose more continuity and flexibility. The Hierarchical Taxonomy of Psychopathology (HiTOP; Kotov et al. 2017; Ruggero et al. 2019) postulates a dimensional characterisation using data‐driven approaches resulting in a hierarchy (symptom components, maladaptive traits, syndromes, spectra and super‐spectra; Kotov et al. 2020; Krueger et al. 2021; Watson et al. 2022). HiTOP suggests conditions share higher‐order dimensions (Kotov et al. 2020; Krueger et al. 2018), which may help explain overlap at (poly‐)genetic (Craddock and Owen 2010; Jones et al. 2018; Tackett et al. 2013), biological (Janiri et al. 2020; Stein et al. 2021), and symptom levels (Borsboom 2017; Caspi et al. 2014; Nelson et al. 2017; Reininghaus et al. 2019, 2016). This highlights the need for more parsimonious models to investigate shared mechanisms and correlates across psychopathology.

Clinical staging distinguishes psychosis onset, progression, and persistence into distinct stages by characterising symptom severity, specificity, and extent of induced disability (Shah et al. 2020; McGorry et al. 2006, 2010). Early stages (1a, 1b) involve non‐specific, mild symptoms and modest impairment that crystallise into more specific syndromes in later stages (2–4) marked by heightened burden, clearer and recognisable symptom patterns, stability, and comorbidity (Cross et al. 2014; Hickie et al. 2013). Staging models can thus help identify individuals at risk of stage progression, such as individuals at ultra‐high risk (UHR) for psychosis (Yung et al. 2005).

Research supports an ‘extended psychosis phenotype’ (Van Os and Linscott 2012; Van Os and Reininghaus 2016) sharing broad demographic, environmental, and familial characteristics with psychosis disorders. Individuals with subclinical psychotic experiences in the general population may develop more severe symptoms, suggesting continuity across different stages of the disorder (phenomenological continuity) that may persist over time (temporal continuity) (Van Os and Linscott 2012; Van Os and Reininghaus 2016). This implies a continuum from milder, subclinical experiences and dysregulation to more pronounced psychotic and affective symptoms, whereby psychotic experiences form part of larger, overlapping symptom patterns (Cornblatt and Carrión 2016).

Taken together, data on the extended psychosis phenotype (Van Os and Linscott 2012; Van Os and Reininghaus 2016), high comorbidity, and underlying symptom dimensions (Kotov et al. 2020; Krueger et al. 2018) support a notion of pluripotency in UHR individuals (Clark et al. 2017). This refers to early expressions of psychosis and affective dysregulation having the potential to progress into various mental health conditions (Johannessen and McGorry 2010; McGorry et al. 2018), much as stem cells differentiate into various cell types. In this sense, early intervention may help prevent more severe illnesses and inform treatment approaches (Scott et al. 2021).

Recent studies using data‐driven methods have identified transdiagnostic phenotypes and multidimensional models (Reininghaus et al. 2019; Quattrone et al. 2019; Shevlin et al. 2017) with distinct dimensions underlying psychosis (e.g., anxiety/depression, positive symptoms, and cognitive symptoms). To elucidate shared vs. non‐shared symptom variance, psychometric methods (e.g., bifactor modelling) have explored whether a general dimension underlies symptoms of psychosis spectrum disorders (Reininghaus et al. 2019, 2016). These models contribute to ongoing debates on co‐occurring symptoms (e.g., affective psychosis vs. psychosis with comorbid affective disorders) and improve our understanding of transdiagnostic symptom dimensions.

While evidence supports a general psychopathology factor above the super‐spectra in FEP and enduring psychosis (Reininghaus et al. 2019), such dimensions have not yet been investigated in youth at UHR for psychosis. Building on previous work (Cowan and Mittal 2021; Cowan et al. 2024), the current study investigates whether (1) a general dimension underlies symptoms in UHR youth and (2) specific symptom dimensions (positive symptoms, negative symptoms, affect, and activation) are justified alongside a general dimension.

## 2 Methods

### 2.1 Study Design and Participants

Baseline data were from the Staged Treatment in Early Psychosis (STEP) study (Hartmann et al. 2022; McGorry et al. 2023; Nelson et al. 2018), investigating sequential treatment for help‐seeking youth at UHR for psychosis. An initial 1343 help‐seeking UHR adolescents and young adults (12–25 years) were recruited from primary (Headspace) and secondary/tertiary (Orygen Youth Health) mental health services in Melbourne, Australia, between April 2016 and January 2019. Of these, 403 participants did not meet inclusion criteria, and 598 individuals declined to participate or did not provide consent, resulting in a final sample of 342 youth (Hartmann et al. 2022; McGorry et al. 2023; Nelson et al. 2018). Participants were eligible if they had sufficient command of the English language, could provide consent, and met UHR criteria according to the Comprehensive Assessment of At‐Risk Mental States (CAARMS) (Yung et al. 2005). Exclusion criteria were: history of a psychotic episode lasting > 1 week, intoxication‐related Attenuated Psychotic Symptoms (APS) (i.e., symptoms disappearing after the intoxication period), psychotic symptoms due to organic causes, metabolic, endocrine or other physical illnesses, developmental disorders, and IQ under 70 (Hartmann et al. 2022; McGorry et al. 2023; Nelson et al. 2018). While cognitive dimensions are central to psychosis risk, developmental disorders were excluded to avoid conflating static neurodevelopmental impairments with dynamic UHR symptoms.

### 2.2 Measures

#### 2.2.1 Socio‐Demographic Characteristics

Data on age, education, gender, country of birth, and migration status were collected (Hartmann et al. 2022; McGorry et al. 2023; Nelson et al. 2018). Education was recoded into primary, secondary, and further education (vocational/university). Parental migration status was recoded to represent whether one or both parents were native Australians or migrants.

#### 2.2.2 UHR Status

The CAARMS (Yung et al. 2005), Social and Occupational Functioning Assessment Scale (SOFAS) (Goldman et al. 1992), Structured Clinical Interview for the DSM‐5 (SCID‐II) schizotypal personality disorder (First et al. 2015), and Family History Index (FHI) determined UHR status (Hartmann et al. 2022). Clinical assessments were conducted by research assistants. UHR status categories were APS, Brief Limited Intermittent Psychotic Symptoms (BLIPS), and/or a vulnerability trait (schizotypal personality diagnosis or first‐degree relative with psychosis).

#### 2.2.3 Psychopathology

The 24‐item Brief Psychiatric Rating Scale (BPRS) (Overall and Gorham 1962; Ventura et al. 1993), a semi‐structured and well‐established clinician‐rated diagnostic instrument, assessed psychopathology with interview‐ and observation‐based items on a 7‐point Likert scale from *not present* (1) to *extremely severe* (7). The lower end of the BPRS scale (2: *very mild*, 3: *mild*) captures subclinical symptoms with no or limited impairment in functioning. Item scores above 4 are considered clinically relevant (Lukoff et al. 1986). BPRS validity and reliability have been demonstrated (Hofmann et al. 2022) and reported in UHR samples (Paetzold et al. 2021). A four‐factor structure consisting of *positive symptoms* (e.g., hallucinations, suspiciousness), *negative symptoms* (e.g., blunted affect, motor retardation), *affect* (e.g., anxiety, guilt, depression), and *activation* (e.g., excitement, motor hyperactivity) was chosen based on previous literature (Dazzi et al. 2016; Meneghelli et al. 2022). This structure aligns with emerging conceptual frameworks, including the broad At‐Risk Mental State (ARMS) perspective and dimensional approaches such as the HiTOP. Diagnoses were determined using the SCID‐I (First et al. 2015).

### 2.3 Statistical Analyses

Item factor analysis (Gibbons and Hedeker 1992; McDonald 2013) for model estimation was conducted using Mplus (Version 8) (Muthén and Muthén 2010), with full‐information maximum likelihood (MLR) estimator with robust SEs. Data were assumed to be missing at random. Three models were compared: a unidimensional model for general psychopathology (Model A), a multidimensional model with four correlated factors (i.e., positive, negative, affect, and activation) (Dazzi et al. 2016) (Model B), and a bifactor model with one general factor and four orthogonal specific factors (Model C). Fit was evaluated using log‐likelihood, the Akaike information criterion (AIC), Bayesian information criterion (BIC), and the Sample‐Size Adjusted BIC (SABIC), with lower values indicating better model fit, as well as on their interpretability and utility for the study aims (Kass and Wasserman 1995). Estimate model parameters and goodness‐of‐fit using the diagonally Weighted Least Squares estimator, mean and variance adjusted (WLSMV), were examined with the root mean square error of approximation (RMSEA) (Steiger 1990) with values at < 0.10 for acceptable and < 0.05 for very good fit, the weighted root mean square residual (WRMR) with a recommended value of ≤ 1.00, and, to assess improvement in fit relative to a saturated model, the Comparative Fit Index (CFI) (Bentler 1990) and the Tucker–Lewis index (TLI) (Bentler and Bonett 1980; Tucker and Lewis 1973) both with values of > 0.90 for acceptable and > 0.95 for very good fit (Brown 2015; Hu and Bentler 1999). To cautiously compare the bifactor (Model C) and multidimensional models (Model B) in terms of predictive utility, we examined the association of factor scores of general and specific dimensions with the number and type of DSM‐5 diagnoses and social functioning (Table S5).

## 3 Results

### 3.1 Sample

Basic sample characteristics (*N* = 342) are summarised in Table 1 (for more details, see Hartmann et al. 2022). The mean age was 17.7 years (range: 12–25), and 57.9% were female. Most participants (85%) had at least one non‐psychotic DSM‐5 diagnosis (Table 2), with anxiety and mood disorders being the most common (< 60%), followed by substance‐use disorders (16%). A majority (85.4%) met criteria for APS, and 11.7% were categorised under combinations of APS, BLIPS, and trait vulnerability groups.

**TABLE 1:** Socio‐demographic characteristics of STEP sample (*N* = 342).

	Mean/count
Age (years, mean ± SD)	17.2 ± 3.2
Gender, *N* (%)	
Male	144 (42.2)
Female	198 (57.9)
Region of birth, *N* (%)	
Australia	305 (89.4)
Asia	15 (4.1)
Europe	8 (2.4)
New Zealand	5 (1.5)
Other	9 (2.6)
Current accommodation, *N* (%)	
House/apartment with family of origin	268 (78.4)
Rented room/apartment/house	51 (14.9)
Owned apartment/house	4 (1.2)
Other	17 (5.0)
Missing	2 (0.6)
Current relationship status, *N* (%)	
Single/never married	232 (67.8)
Partnered (3 months to 2 years)	60 (17.5)
Partnered (< 3 months)	24 (7.0)
Married/de facto	19 (5.6)
Separated/divorced	2 (0.6)
Other	5 (1.5)
Currently in education, *N* (%)	
No	95 (27.8)
Yes	245 (71.6)
Missing	2 (0.6)
Highest level of education, *N* (%)	
Primary school	1 (0.3)
Secondary school	261 (76.5)
Further education	67 (19.7)
Other	10 (2.9)
Missing	3 (0.8)
Current employment, *N* (%)	
Unemployed	218 (63.7)
Casual paid employment	70 (20.5)
Part‐time paid employment	27 (7.9)
Full‐time paid employment	12 (3.5)
Casual unpaid employment	8 (2.3)
Part‐time unpaid employment	2 (0.6)
Missing	5 (1.5)
Parental migration, *N* (%)	
Both parents native Australian	181 (52.9)
One parent native Australian	67 (19.6)
Both parents immigrated to Australia	64 (18.7)
Missing	30 (8.8)

**TABLE 2:** Clinical characteristics of STEP sample (*N* = 342).

	Mean/count
Comorbidity, *N* (%)	215 (70)
Current diagnosis, *N* (%)	
No diagnosis	45 (13.2)
Anxiety, dissociative, stress‐related, somatoform and other non‐psychotic mental disorders	225 (65.8)
Mood disorders	207 (60.5)
Mental and behavioural disorders due to psychoactive substance use	54 (15.8)
Behavioural syndromes associated with physiological disturbances and physical factors	30 (8.8)
Behavioural and emotional disorders with onset usually occurring in childhood and adolescence	26 (7.6)
Borderline personality disorder	17 (5.0)
Pervasive and specific developmental disorders	7 (2.0)
Schizotypal disorder	6 (1.8)
Obsessive‐compulsive personality disorder	1 (0.3)
Other	1 (0.3)
Missing	15 (4.4)
UHR subgroups, *N* (%)	
Trait vulnerability	9 (2.6)
APS	292 (85.4)
BLIPS	1 (0.3)
Combined	40 (11.7)
Other symptoms, mean (±SD)	
General psychopathology (BPRS)	44.6 ± 8.7
Missing^a^	2
Negative symptoms (SANS)	18.4 ± 11.2
Missing^a^	1
Depressive symptoms (MADRS)	23.5 ± 9.9
Missing^a^	5

Abbreviations: APS, Attenuated Psychosis Symptoms; BLIPS, Brief Intermittent Psychotic Symptoms, BPRS, Brief Psychiatric Rating Scale; MADRS, Montgomery‐Åsberg Depression Rating Scale; SANS, Scale for the Assessment of Negative Symptoms; UHR, Ultra‐High Risk for Psychosis.

^a^ Cases with missing data across all items of the scale.

### 3.2 Model Comparison

Table 3 shows the model fit statistics for all three models. In line with established guidelines for information criteria and best practice for model selection, we interpreted differences in AIC, BIC, and SABIC greater than 10 as providing *strong* evidence of fit (Raftery 1995; Reise et al. 2010). The bifactor model with one general and four specific factors (i.e., positive, negative, affect, and activation) showed the best relative fit based on AIC, BIC, and SABIC and factor loadings compared to the unidimensional (Table S1) and multidimensional (Table S2) models. However, additional absolute fit indices (CFI, TLI, and RMSEA) and factor loadings computed with another estimator, WLSMV, demonstrated a different pattern of findings (Tables S3 and S4), with none of the models emerging to have adequate absolute fit (Table 3).

**TABLE 3:** Model fit statistics for unitary (unidimensional), multidimensional and bifactor models of psychosis based on BPRS symptom ratings.

	Full information fit statistics^a^								
	LL^b^, ^c^	FP^b^, ^c^	AIC^b^	BIC^b^	SABIC^b^	WRMR^c^	RMSEA^c^	CFI^c^	TLI^c^
Unidimensional (unitary) model (Model A)	−7963.7	133	16 193.4	16 702.6	16 280.7	2.358	0.124	0.625	0.590
Multidimensional model with four correlated specific factors (Model B)	−7735.0	139	15 748.0	16 280.2	15 839.3	1.760	0.090	0.809	0.785
Bifactor model with one general factor and four specific symptom factors (Model C)	−7663.0	157	15 640.0	16 241.2	15 743.1	1.465	0.078	0.867	0.839

*Note:* Model C provides the best model fit, as indicated by lower BIC, AIC, and SABIC compared to other models and a low RMSEA (< 0.10 for acceptable and < 0.05 for very good fit), and CFI/TLI at threshold (> 0.90 for acceptable and > 0.95 for very good fit). The WRMR of Model C is closest to the recommended value (≤ 1.00). All response vectors with at least one response were analysed (*N* = 340).

Abbreviations: AIC, Akaike information criterion; BIC, Bayesian information criterion; BPRS, Brief Psychiatric Rating Scale; CFI, comparative fit index; FP, free parameters; LL, log‐likelihood; RMSEA, root mean square error of approximation; SABIC, sample size‐adjusted Bayesian information criterion; TLI, Tucker–Lewis Index; WRMR, weighted root mean square residual.

^a^ A difference of 10 in AIC, BIC, and SABIC is considered important.

^b^ Fit statistics calculated using the full‐information maximum likelihood (MLR) estimator with robust SEs.

^c^ Fit statistics calculated with the weighted least squares mean and variance adjusted (WLSMV) estimator.

### 3.3 General and Specific Dimensions

Factor loadings in the bifactor model were heterogeneous in magnitude across dimensions (Table 4). Most BPRS items loaded onto both the general factor *and* one specific factor. The general factor showed moderate to strong loadings on items not directly related to UHR, such as *somatic concern* (factor loading of 0.38, *p* < 0.001), *disorientation* (factor loading of 0.72, *p* < 0.001), *self‐neglect* (factor loading of 0.50, *p* < 0.001), *tension* (factor loading of 0.48, *p* < 0.001) and *mannerisms and posturing* (factor loading of 0.44, *p* < 0.01).

**TABLE 4:** Factor loadings in bifactor model with a general factor and four specific factors based on BPRS symptom ratings.

BPRS items	General	Positive symptoms	Negative symptoms	Affect	Activation
Grandiosity	−0.10	0.55**			
Suspiciousness	0.20*	0.37**			
Hallucinations	0.17*	0.19*			
Unusual thought content	0.18*	0.74**			
Bizarre behaviour	0.22*	0.17			
Conceptual disorganisation	0.68**	0.16			
Self‐neglect	0.50**		0.004**		
Disorientation	0.72**		0.06**		
Blunted affect	0.32**		0.87**		
Emotional withdrawal	0.38**		0.88**		
Motor retardation	0.34**		0.67**		
Uncooperativeness	0.49**		0.47**		
Somatic concern	0.38**			0.002**	
Anxiety	0.24*			0.46**	
Depression	0.30**			0.80**	
Suicidality	0.24*			0.69**	
Guilt feelings	0.36**			0.34**	
Hostility	0.23*				0.01**
Elevated mood	−0.33*				0.59**
Tension	0.48**				0.51**
Excitement	0.01				0.81**
Distractibility	0.48**				0.39**
Motor hyperactivity	0.39**				0.80**
Mannerisms and posture	0.44*				0.47**
Relative Omega (ω)	0.63	0.72	0.54	0.67	0.82

Abbreviation: BPRS, Brief Psychiatric Rating Scale.

* *p* < 0.01.

** *p* < 0.001.

Some items, particularly in negative (e.g., blunted affect, emotional withdrawal, and motor retardation) and positive symptoms dimensions (e.g., grandiosity, unusual thought content), significantly loaded more strongly on their respective specific factor (0.55–0.88) than on the general factor (Table 4), suggesting domain‐specific variance. These items seem to reflect distinct clinical phenomena rather than being mere expressions of general severity. Within the affective dimension, variance in responses on the BPRS items anxiety (0.46), depression (0.80), and suicidality (0.69) was all marginally better captured by the affect‐specific factor. Guilt was not clearly associated with either the general (0.36) or affective (0.34) factors, likely reflecting shared variance between general distress and affective experiences. In the activation dimension, elevated mood, motor hyperactivity, and excitement loaded more strongly on the specific factor (0.59–0.81), while tension and mannerisms showed similar loadings (0.44–0.51) to both. Elevated mood loaded negatively on the general factor (−0.33), perhaps linked to distinct, potentially protective, manic‐like features or hypomanic states that do not align with general distress. The bifactor model (Table 4) tentatively demonstrated stronger discriminatory ability compared to the multidimensional model (Table S2). While the multidimensional model showed higher factor loadings overall, the bifactor model, with moderate to high loadings (i.e., > 0.50) on both general and specific factors as well as moderate cross‐loadings (e.g., tension, distractibility, and uncooperativeness), allowed for a differentiation of shared and specific variance.

The general factor (*ω* = 0.63) accounted for a moderate amount of total variance, while specific factors, such as activation (*ω* = 0.82) and positive symptoms (*ω* = 0.72), explained a larger proportion within their respective item subsets (Table 4). This omega value suggests a common underlying dimension that contributes to the variability across all items, but it may not be as dominant as some specific factors, ranging from 0.54 to 0.82. Overall, while there is a ‘common underlying factor’ (general factor), as per design in orthogonal bifactor modelling, each item subset also taps into distinct sources of variance (specific factors). Moreover, the bifactor model showed greater predictive value across external validators (e.g., social functioning and number of diagnoses) compared to the multidimensional model (Table S5).

## 4 Discussion

### 4.1 Primary Findings

This study tentatively supports a pluripotent risk state at the phenotypic level in a sample of young individuals at UHR for psychosis. The formation of specific symptom dimensions (i.e., positive symptoms, negative symptoms, affect, and activation) is, on a relative level, justified in addition to a general dimension. Compared to unidimensional and multidimensional alternatives, the bifactor model with one general factor and four specific factors provided the best relative, but not absolute, model fit and interpretability. Thus, given how variance is distributed in bifactor modelling, the general factor seemed not solely influenced by the UHR state for psychosis but rather by a broader range of symptoms as captured by the BPRS‐24. This suggests that the BPRS may index both a general dimension and several more specific dimensions, cautiously reflecting the pluripotency underlying psychological features in the UHR sample. Findings of ‘common ground’ across the risk syndrome in this heterogeneous sample, with various comorbidities, would lend further evidence to support the notion of a transdiagnostic phenotype of psychopathology in youth.

### 4.2 Methodological Considerations

The strength of our study is that we used item factor analysis to examine the dimensionality of psychopathology in UHR youth, identifying variance driven by both a general psychosis dimension and specific dimensions, consistent with recent efforts to broaden the UHR concept (Hartmann et al. 2019). We also assessed psychopathological features not specific to psychosis with a widely used measure, the BPRS‐24, to capture transdiagnostic symptom dimensions in the sample. However, several limitations should be noted. First, there was a high rate of comorbidity. Many participants were diagnosed with more than one (and up to seven) current mental health conditions based on diagnostic instruments such as the SCID‐I (First et al. 2015). This comorbidity may in part account for the identified general factor and not readily generalise to individuals at UHR for psychosis without any comorbidity. It may, however, also be characteristic of the broad level of impairment and distress commonly seen in individuals at UHR for psychosis and illustrative of their help‐seeking behaviour (van Os and Guloksuz 2017). Comorbidity mostly included the simultaneous presence of anxiety alongside non‐psychotic, affective (mood) disorders and substance use‐related disorders. These conditions are highly prevalent within the APS subgroup among individuals at UHR for psychosis, contributing to comorbid common mental disorders in this population (Fusar‐Poli et al. 2016). Further, the current study did not include, or at least not formally identify, individuals at UHR for severe mental disorders other than psychosis, which would have helped to disentangle overlap with, or independence from, other important UHR states. Finally, it has been noted that bifactor models may be hard to interpret and overfit the data (Bonifay et al. 2017; Greene et al. 2019). However, the bifactor model allowed us to address dimensionality issues that may emerge when the conceptual breadth of a construct cannot be fully determined (i.e., is unidimensional and multidimensional at the same time) (Reise et al. 2007), which is likely the case for UHR psychosis. Moreover, we did not only focus on model fit indices but also on the interpretability of models and their clinical utility. Given that the multidimensional model with correlated factors is nested within the bifactor model, it can encompass the multidimensional model with correlated factors as a special case. This makes the bifactor model a more inclusive framework and, as a result, may provide a better fit to the data compared to the multidimensional model when the same constraints are applied, given its ability to account for model misspecification.

We recognise the potential risk of overfitting and artificial fit improvements in our analyses and carefully consider the theoretical implications of any post hoc modifications (MacCallum et al. 1992; Muthén and Asparouhov 2012; Preacher and MacCallum 2003), ensuring that such changes are not purely data‐driven but are also justifiable within the broader framework. In our understanding, this balance helps to maintain the model's theoretical integrity and enhances the likelihood that the model's findings will generalise to other samples and settings and not reflect an artificial increase in model fit. Several factors may have contributed to the modest absolute model fit observed across structures. Symptom heterogeneity in UHR individuals may have resulted in residual correlations that are not fully accounted for by latent factors. Additionally, item‐level cross‐loading, particularly among non‐specific symptoms, may slightly attenuate overall fit despite conceptually coherent factor structures. While the present study focused on confirmatory approaches, future research should incorporate longitudinal designs and consider alternative modelling frameworks, such as network analysis or exploratory structural equation modelling (ESEM), to better capture the complexity and dynamics of symptom expression in this population.

While we recognise the importance of parsimony in model selection, the theoretical advantages and the inclusive nature of the bifactor framework, as evidenced in our analyses, justify its complexity. This complexity allows for a more nuanced understanding of the psychological constructs in question, as per our theoretical framework, and may help untangle the complexity often encountered when trying to fit multidimensional data into a unidimensional model. In such cases, the bifactor model helps clarify the underlying psychological construct being measured (Reise 2012). Hence, the bifactor model was a suitable model to be compared with unidimensional and multidimensional models for addressing dimensionality issues and generating early dimensional phenotypes in the UHR state for psychosis.

### 4.3 Comparison With Previous Research

Using item factor analyses, we advanced previous research on the dimensionality of psychosis and early psychopathology by identifying latent general and specific dimensions as key contributors to mental health symptoms in individuals at UHR for psychosis. Intriguingly, findings across different samples consistently suggest that the bifactor model, which includes one general and four specific factors, provided better fit and interpretability compared to alternative models (Reininghaus et al. 2019, 2016, 2013; Quattrone et al. 2019).

Recent dimensional research has postulated an underlying general factor in psychosis spectrum disorders (Reininghaus et al. 2019, 2016, 2013; Quattrone et al. 2019). However, this was never investigated in youth with UHR for psychosis. Almost all BPRS items loaded onto both the general factor and specific psychosis dimensions in UHR youth, echoing prior findings of a general factor in the general population (Shevlin et al. 2017), in individuals with acute FEP or second episode psychosis (Reininghaus et al. 2013) and schizophrenia, schizoaffective disorder, or psychotic bipolar I (Reininghaus et al. 2019, 2016), suggesting a pluripotent phenotype. It has been previously proposed that a general factor of psychopathology (Caspi et al. 2014; Lahey et al. 2012, 2011), as the overarching transdiagnostic super‐spectrum in HiTOP, may be an important target for prevention (Forbes et al. 2019, 2021). The current study identifies a general factor as a putative dimensional representation of a pluripotent risk state in UHR for psychosis and, hence, places the latter on the super‐spectrum within hierarchical models of psychopathology. More generally, it connects clinical staging models with HiTOP, which reflects an important step in linking dimensional models and longitudinal emergence of early phenotypes in psychopathology. In our sample, we observed a relatively strong general factor alongside weaker, yet interpretable, specific dimensions (e.g., affect and activation), which may reflect the diffuse and heterogeneous nature of early psychopathology. This aligns with clinical staging models and prior research suggesting that broad, transdiagnostic symptom expression is more prominent in early illness stages, while more differentiated symptom clusters emerge later (Hartmann et al. 2019; McGorry 2014). Further longitudinal work is warranted to capture dimensional representations of clinical stages and investigate risk and resilience mechanisms and their relation to specific and general dimensions to potentially shed light on why some UHR individuals go on to develop early and enduring psychosis. While our cross‐sectional data do not allow us to track these emergences directly, future longitudinal research is warranted to examine whether and how UHR bifactor structures reorganise across stages of illness and determine whether early general factors predict later clinical outcomes or transitions into full‐threshold disorders. This also raises the question of whether early interventions should target broad affective and cognitive dysregulation, rather than narrowly defined syndromes, before more stable diagnostic patterns consolidate.

Early symptoms of mental disorder may not represent a fixed trajectory to certain diagnoses, and may even evolve into a range of different psychiatric syndromes (McGorry 2014, 2013). In a retrospective study, Shah et al. (2017) found that almost one‐third of their sample with FEP did not experience sub‐threshold psychotic symptoms before developing psychosis, and that depression, anxiety, and low functioning were the most common early symptoms. This supports recent efforts advocating a broader approach to identifying and treating youth at risk, including Clinical High At‐Risk Mental States (CHARMS) (Hartmann et al. 2019), which widens the UHR state to include transdiagnostic at‐risk mental states beyond APS (Hartmann et al. 2019). Given that UHR individuals have elevated risk for exit syndromes beyond psychosis, including mood, anxiety, substance use and personality disorders (Fusar‐Poli et al. 2014; Lin et al. 2015; Rutigliano et al. 2016), CHARMS may be especially valuable. Clinical staging models consequently align with the prospect of pluripotency in early clinical phenotypes and allow for comorbid outcomes (McGorry et al. 2006, 2018; Hickie et al. 2013). The factor loadings of somatic concern (0.38) and disorientation (0.72) onto the general factor may reflect early broad prodromal dysregulation that is not specific to psychosis per se, but signals heightened pluripotent vulnerability. This dimensional representation requires further longitudinal testing to determine whether non‐specific symptoms are associated with distinct trajectories of clinical outcomes. Our findings of a general factor in the UHR state may help move the needle forward on developing novel approaches to identifying individuals at risk for developing mental health problems and promoting a transdiagnostic view on youth mental health care.

In contrast to previous research on psychosis dimensions, most individuals in our sample were diagnosed with other, non‐psychotic disorders. This may have facilitated the disentanglement with other important spectra of psychopathology (Reininghaus et al. 2016; Böhnke and Croudace 2015) and suggests that the UHR status may not be limited to psychosis (Hartmann et al. 2019). The BPRS‐24 (Overall and Gorham 1962; Ventura et al. 1993) was thus well suited to capturing the wide symptom range experienced by young individuals at UHR for psychosis. Moreover, in the current study, a UHR subsample (*n* = 40) with no current mental illness suggests that the general factor underlying the UHR state may not be dependent on pre‐existing mental health conditions. Instead, this general factor may represent a transdiagnostic vulnerability to developing psychopathology that is present in both individuals with and without other mental health conditions. Each item's discriminatory capacity on its respective factor is pivotal. Certain items demonstrated stronger discriminatory ability on the general factor rather than on specific factors (i.e., *bizarre behaviour*, *conceptual disorganisation*, *self‐neglect*, *disorientation*, *uncooperativeness*, *somatic concern*, *guilt*, *hostility,* and *distractibility*). The bifactor model, characterised by its imposed orthogonal structure, allows us to discern the presence of an ‘essential unidimensionality’ (Reise et al. 2007), and whether items exhibit discriminatory ability beyond this general dimension onto specific factors. This analysis offers insights into the relative informativeness of items on either the general or specific factors, or lack thereof if statistically non‐significant and limited in magnitude.

Characterising this general factor further could potentially support earlier detection and intervention for individuals at risk of developing psychopathology, even if they do not have other, pre‐existing diagnoses of mental health conditions. Distinguishing between the general psychopathology factor (transdiagnostic risk) and specific dimensions (e.g., positive symptoms and affect) may enable more targeted interventions. This may entail broad interventions (general factor), such as stress reduction or social skills training, for shared vulnerability pathways, or specific approaches, such as CBT for positive symptoms or emotion regulation for affect, for specific dimensions (Choate et al. 2023; Doborjeh et al. 2024). Notably, items that loaded most strongly on the general factor in the bifactor model were *conceptual disorganisation*, *disorientation,* and *self‐neglect*. These non‐specific symptoms in UHR individuals may play an important role in risk trajectories by increasing the frequency and intensity of subthreshold experiences, delaying symptom specificity, and contributing to declines in social and functional domains, all of which are key predictors of later clinical outcomes in broad at‐risk populations (Yung et al. 2019).

Future research should continue to corroborate the clinical validity of the assessed dimensions in UHR individuals through associations with established primary DSM‐5 diagnoses as well as other clinical, social, and demographic variables. Recent empirical work demonstrated that dimensional ratings of psychosis symptom severity provided greater predictive value for functional outcomes in youth at clinical high risk than traditional categorical risk status, highlighting the advantages of dimensional models for both research and clinical practice (Phalen et al. 2022). Such exploration may help determine whether dimensions are more predictive and phenotypic of mental disorder than categorical classifications.

## 5 Conclusions

The findings offer some evidence for a shared general dimension across the psychosis risk syndrome, tentatively indicating a pluripotent risk state, while simultaneously acknowledging the contribution of domain‐specific factors. However, no strong statistical evidence supported the bifactor model over other models based on absolute fit indices. The general factor found in our UHR sample suggests a pluripotent at‐risk state, rather than a p‐factor analogue or psychosis super‐spectrum. By identifying a single general factor, the bifactor model loadings indicate a common underlying vulnerability for psychosis among individuals at UHR. This suggests that previously observed risk factors in this group may not be confined to a singular domain but rather manifest across various ‘symptom components’ (in HiTOP speak), reflecting a broader susceptibility to diverse mental health symptoms. These findings shed light on symptom dimensionality in UHR youth and underscore the need to further investigate transdiagnostic phenotypes at developmentally early stages of psychopathology. Refining our knowledge of mental health conditions' dimensional nature at these stages could help bridge clinical staging models with HiTOP as an important step in linking dimensional structure and longitudinal emergence of early phenotypes and, in turn, intervening earlier along the continuum of psychopathology, such as metacognitive training for attenuated positive symptoms or emotion regulation therapy for affective dimensions. Our findings contribute to converging data on transdiagnostic phenotypes and highlight the importance of further exploring pluripotency in mental health conditions.

## Conflicts of Interest

The authors declare no conflicts of interest.

## Supporting information


**Table S1:** Factor loadings in unidimensional model with a general factor based on BPRS symptom ratings.

**Table S2:** Factor loadings in multidimensional model with four specific factors based on BPRS symptom ratings.

**Table S3:** Factor loadings in bifactor model with a general factor and four specific factors based on BPRS symptom ratings (WLSMV estimator).

**Table S4:** Factor loadings in multidimensional model with four specific factors based on BPRS symptom ratings (WLSMV estimator).

**Table S5:** Associations of factor scores from multidimensional and bifactor models with social functioning, diagnoses, and number of diagnoses.

## Data Availability

The data that support the findings of this study are available on request from the corresponding author. The data are not publicly available due to privacy or ethical restrictions.
