# Supplementary material for: Transdiagnostic Symptom Dimensions in Individuals at Ultra‐High Risk for Psychosis: Towards Dimensional Representations of Pluripotent Risk
Source: Early Interv Psychiatry. 2025 Aug 21;19(8):e70086. doi: 10.1111/eip.70086 (PMC12368483; doi:10.1111/eip.70086)
Supplement: Supplementary file 1 — Table S1: Factor loadings in unidimensional model with a general factor based on BPRS symptom ratings. [file EIP-19-0-s005.docx]

**Table S1.** Factor Loadings in Unidimensional Model with a General Factor based on BPRS Symptom Ratings

| **BPRS items** | **General** |
| --- | --- |
| Grandiosity | -0.05 |
| Suspiciousness | 0.14^*^ |
| Hallucinations | 0.13^*^ |
| Unusual thought content | 0.17^*^ |
| Bizarre behaviour | 0.08 |
| Conceptual disorganization | 0.26^*^ |
| Self-neglect | 0.23^**^ |
| Disorientation | 0.36^**^ |
| Blunted affect | 0.93^**^ |
| Emotional withdrawal | 0.94^**^ |
| Motor retardation | 0.77^**^ |
| Uncooperativeness | 0.61^**^ |
| Somatic concern | 0.08 |
| Anxiety | 0.18^**^ |
| Depression | 0.40^**^ |
| Suicidality | 0.29^**^ |
| Guilt feelings | 0.22^**^ |
| Hostility | 0.08 |
| Elevated mood | -0.39^**^ |
| Tension | 0.16^*^ |
| Excitement | -0.47^**^ |
| Distractibility | 0.06 |
| Motor hyperactivity | -0.06 |
| Mannerisms and posture | 0.35^*^ |

**Note:** BPRS – Brief Psychiatric Rating Scale

∗*p* < .05; ∗∗*p* < .001
