# Supplementary material for: Transdiagnostic Symptom Dimensions in Individuals at Ultra‐High Risk for Psychosis: Towards Dimensional Representations of Pluripotent Risk
Source: Early Interv Psychiatry. 2025 Aug 21;19(8):e70086. doi: 10.1111/eip.70086 (PMC12368483; doi:10.1111/eip.70086)
Supplement: Supplementary file 2 — Table S2: Factor loadings in multidimensional model with four specific factors based on BPRS symptom ratings. [file EIP-19-0-s001.docx]

**Table S2.** Factor Loadings in Multidimensional Model with Four Specific Factors based on BPRS Symptom Ratings

| **BPRS items** | **Positive symptoms** | **Negative**  **symptoms** | **Affect** | **Activation** |
| --- | --- | --- | --- | --- |
| Grandiosity | 0.34^*^ |  |  |  |
| Suspiciousness | 0.53^**^ |  |  |  |
| Hallucinations | 0.31^**^ |  |  |  |
| Unusual thought content | 0.59^**^ |  |  |  |
| Bizarre behaviour | 0.30^*^ |  |  |  |
| Conceptual disorganization | 0.45^**^ |  |  |  |
| Self-neglect |  | 0.21^*^ |  |  |
| Disorientation |  | 0.33^**^ |  |  |
| Blunted affect |  | 0.93^**^ |  |  |
| Emotional withdrawal |  | 0.95^**^ |  |  |
| Motor retardation |  | 0.76^**^ |  |  |
| Uncooperativeness |  | 0.61^**^ |  |  |
| Somatic concern |  |  | 0.17^*^ |  |
| Anxiety |  |  | 0.54^**^ |  |
| Depression |  |  | 0.81^**^ |  |
| Suicidality |  |  | 0.72^**^ |  |
| Guilt feelings |  |  | 0.50^**^ |  |
| Hostility |  |  |  | 0.14^*^ |
| Elevated mood |  |  |  | 0.24^*^ |
| Tension |  |  |  | 0.71^**^ |
| Excitement |  |  |  | 0.62^**^ |
| Distractibility |  |  |  | 0.52^**^ |
| Motor hyperactivity |  |  |  | 0.96^**^ |
| Mannerisms and posture |  |  |  | 0.56^**^ |

**Note:** BPRS – Brief Psychiatric Rating Scale

∗*p* < .05; ∗∗*p* < .001
