# Supplementary material for: Transdiagnostic Symptom Dimensions in Individuals at Ultra‐High Risk for Psychosis: Towards Dimensional Representations of Pluripotent Risk
Source: Early Interv Psychiatry. 2025 Aug 21;19(8):e70086. doi: 10.1111/eip.70086 (PMC12368483; doi:10.1111/eip.70086)
Supplement: Supplementary file 3 — Table S3: Factor loadings in bifactor model with a general factor and four specific factors based on BPRS symptom ratings (WLSMV estimator). [file EIP-19-0-s003.docx]

**Table S3.** Factor Loadings in Bifactor Model with a General Factor and Four Specific Factors based on BPRS Symptom Ratings (WLSMV estimator)

| **BPRS items** | **General** | **Positive symptoms** | **Negative**  **symptoms** | **Affect** | **Activation** |
| --- | --- | --- | --- | --- | --- |
| Grandiosity | 0.00 | 0.44^*^ |  |  |  |
| Suspiciousness | 0.33^**^ | 0.25^*^ |  |  |  |
| Hallucinations | 0.24^**^ | 0.13 |  |  |  |
| Unusual thought content | 0.31^**^ | 0.94^**^ |  |  |  |
| Bizarre behaviour | 0.36^**^ | 0.08 |  |  |  |
| Conceptual disorganization | 0.72^**^ | 0.00 |  |  |  |
| Self-neglect | 0.47^**^ |  | 0.05 |  |  |
| Disorientation | 0.66^**^ |  | 0.12 |  |  |
| Blunted affect | 0.31^**^ |  | 0.86^**^ |  |  |
| Emotional withdrawal | 0.33^**^ |  | 0.91^**^ |  |  |
| Motor retardation | 0.19 |  | 0.73^**^ |  |  |
| Uncooperativeness | 0.39^**^ |  | 0.49^**^ |  |  |
| Somatic concern | 0.43^**^ |  |  | -0.06 |  |
| Anxiety | 0.33^**^ |  |  | 0.40^**^ |  |
| Depression | 0.41^**^ |  |  | 0.75^**^ |  |
| Suicidality | 0.34^**^ |  |  | 0.64^**^ |  |
| Guilt feelings | 0.44^**^ |  |  | 0.32^**^ |  |
| Hostility | 0.26^**^ |  |  |  | 0.02 |
| Elevated mood | -0.31^*^ |  |  |  | 0.58^**^ |
| Tension | 0.49^**^ |  |  |  | 0.54^**^ |
| Excitement | -0.01 |  |  |  | 0.82^**^ |
| Distractibility | 0.41^**^ |  |  |  | 0.43^**^ |
| Motor hyperactivity | 0.37^**^ |  |  |  | 0.83^**^ |
| Mannerisms and posture | 0.58^*^ |  |  |  | 0.48^**^ |
|  |  |  |  |  |  |

**Note:** BPRS – Brief Psychiatric Rating Scale, WLSMV - Weighed Least Squares Mean and Variance adjusted

∗*p* < .01; ∗∗*p* < .001
