# Supplementary material for: Transdiagnostic Symptom Dimensions in Individuals at Ultra‐High Risk for Psychosis: Towards Dimensional Representations of Pluripotent Risk
Source: Early Interv Psychiatry. 2025 Aug 21;19(8):e70086. doi: 10.1111/eip.70086 (PMC12368483; doi:10.1111/eip.70086)
Supplement: Supplementary file 4 — Table S4: Factor loadings in multidimensional model with four specific factors based on BPRS symptom ratings (WLSMV estimator). [file EIP-19-0-s002.docx]

**Table S4.** Factor Loadings in Multidimensional Model with Four Specific Factors based on BPRS Symptom Ratings (WLSMV estimator)

| **BPRS items** | **Positive symptoms** | **Negative**  **symptoms** | **Affect** | **Activation** |
| --- | --- | --- | --- | --- |
| Grandiosity | 0.19^*^ |  |  |  |
| Suspiciousness | 0.42^**^ |  |  |  |
| Hallucinations | 0.26^**^ |  |  |  |
| Unusual thought content | 0.43^**^ |  |  |  |
| Bizarre behaviour | 0.43^**^ |  |  |  |
| Conceptual disorganization | 0.70^**^ |  |  |  |
| Self-neglect |  | 0.33^**^ |  |  |
| Disorientation |  | 0.51^**^ |  |  |
| Blunted affect |  | 0.91^**^ |  |  |
| Emotional withdrawal |  | 0.96^**^ |  |  |
| Motor retardation |  | 0.73^**^ |  |  |
| Uncooperativeness |  | 0.58^**^ |  |  |
| Somatic concern |  |  | 0.31^**^ |  |
| Anxiety |  |  | 0.53^**^ |  |
| Depression |  |  | 0.80^**^ |  |
| Suicidality |  |  | 0.70^**^ |  |
| Guilt feelings |  |  | 0.58^**^ |  |
| Hostility |  |  |  | 0.22^**^ |
| Elevated mood |  |  |  | 0.32^**^ |
| Tension |  |  |  | 0.74^**^ |
| Excitement |  |  |  | 0.65^**^ |
| Distractibility |  |  |  | 0.57^**^ |
| Motor hyperactivity |  |  |  | 0.90^**^ |
| Mannerisms and posture |  |  |  | 0.71^**^ |
|  |  |  |  |  |

**Note:** BPRS – Brief Psychiatric Rating Scale, WLSMV - Weighed Least Squares Mean and Variance adjusted

∗*p* < .01; ∗∗*p* < .001
