# Supplementary material for: Transdiagnostic Symptom Dimensions in Individuals at Ultra‐High Risk for Psychosis: Towards Dimensional Representations of Pluripotent Risk
Source: Early Interv Psychiatry. 2025 Aug 21;19(8):e70086. doi: 10.1111/eip.70086 (PMC12368483; doi:10.1111/eip.70086)
Supplement: Supplementary file 5 — Table S5: Associations of factor scores from multidimensional and bifactor models with social functioning, diagnoses, and number of diagnoses. [file EIP-19-0-s004.docx]

**Table S5.** Associations of Factor Scores from Multidimensional and Bifactor Models with Social Functioning, Diagnoses, and Number of Diagnoses

|  |  | **Number of Diagnoses** | |  | **Social Functioning** | |  | **Diagnoses** | | | | |
| --- | --- | --- | --- | --- | --- | --- | --- | --- | --- | --- | --- | --- |
|  |  |  | |  |  | |  | **CMD** | |  | **Other Diagnoses** | |
| **Predictor** | | Coefficient^†^  (95% CI) | *p* |  | Coefficient^†^  (95% CI) | *p* |  | Coefficient^‡^  (95% CI) | *p* |  | Coefficient^‡^  (95% CI) | *p* |
| **Bifactor Model** | | | | | | | | | | | | |
|  | |  |  |  |  |  |  |  |  |  |  |  |
| G Factor | | 0.74  (0.40 - 1.08) | <0.05 |  | -7.67  (-10.80 - -4.54) | <0.001 |  | 1.65  (0.29 - 3.00) | <0.05 |  | 1.98  (0.52 - 4.43) | <0.05 |
| Positive Symptoms | | 0.16  (-0.09 - 0.41) | 0.23 |  | 0.56  (-1.80 - 2.92) | 0.64 |  | 0.027  (-0.92 - 0.98) | 0.96 |  | 0.29  (-0.73 - 1.30) | 0.58 |
| Negative Symptoms | | -0.69  (-1.41 - 0.04) | 0.06 |  | -7.43  (-14.34 - -0.52) | 0.04 |  | 1.40  (-1.45 - 4.26) | 0.33 |  | -0.43  (-3.56 - 2.70) | 0.79 |
| Affect | | 3.65  (2.92 - 4.37) | <0.001 |  | -12.50  (-19.39 - -5.61) | <0.001 |  | 5.92  (3.06 - 8.76) | <0.001 |  | 6.75  (3.64 - 9.86) | <0.001 |
| Activation | | 0.89  (0.02 - 1.75) | <0.05 |  | -2.30  (-10.55 - 6.00) | 0.59 |  | 3.28  (-0.42 - 7.00) | 0.08 |  | 5.37  (1.47 - 9.27) | <0.05 |
|  |  |  |  |  |  |  |  |  |  |  |  |  |
| **Multidimensional Model** | | | | | | | | | | | | |
|  | |  |  |  |  |  |  |  |  |  |  |  |
| Positive Symptoms | | -0.04  (-0.30 - 0.22) | 0.75 |  | 1.55  (-0.67 - 3.77) | 0.17 |  | 0.26  (-0.51 - 1.02) | 0.52 |  | 0.45  (-0.38 - 1.28) | 0.29 |
| Negative Symptoms | | -0.36  (-0.81 - 0.09) | 0.12 |  | -4.36  (-8.27 - -0.45) | <0.05 |  | -0.48  (-1.80 - 0.84) | 0.48 |  | -1.10  (-2.56 - 0.36) | 0.14 |
| Affect | | 1.95  (1.18 - 2.71) | <0.001 |  | -8.84  (-15.44 - -2.23) | <0.05 |  | 2.76  (0.40 - 5.11) | <0.05 |  | 2.47  (-0.06 - 5.00) | 0.05 |
| Activation | | 0.28  (-0.51 - 1.06) | 0.49 |  | -6.59  (-13.50 - 0.30) | 0.06 |  | -0.03  (-2.51 - 2.46) | 0.98 |  | 0.62  (-2.02 - 3.27) | 0.65 |
|  |  |  | |  |  |  |  |  |  |  |  |  |

**Note***:* CI - Confidence Interval, CMD - Common Mental Disorder (ICD-10 F1, F3, F4), Other Diagnoses: ICD-10 codes with less than 50 cases (ICD-10 F2, F5, F6, F8, F9, N9).

Social functioning assessed with the SOFAS (Social and Occupational Functioning Assessment Scale). N=342

^†^Linear Regression Model

^‡^Multinomial Regression Model, Outcome Base (Reference) Category: No Diagnosis
